# Carotid Artery Ultrasound in the (peri-) Arrest Setting—A Prospective Pilot Study

**DOI:** 10.3390/jcm11020469

**Published:** 2022-01-17

**Authors:** Moritz Koch, Matthias Mueller, Alexandra-Maria Warenits, Michael Holzer, Alexander Spiel, Sebastian Schnaubelt

**Affiliations:** 1 Department of Emergency Medicine, Medical University of Vienna, 1090 Vienna, Austria; moritz.koch@meduniwien.ac.at (M.K.); matthias.mueller@meduniwien.ac.at (M.M.); alexandra-maria.warenits@meduniwien.ac.at (A.-M.W.); michael.holzer@meduniwien.ac.at (M.H.); sebastian.schnaubelt@meduniwien.ac.at (S.S.); 2 Vienna Health Care Group, Department of Emergency Medicine, Clinic Ottakring, 1160 Vienna, Austria

**Keywords:** point-of-care ultrasound, carotid ultrasound, emergency medicine, cardiac arrest, cardiopulmonary resuscitation

## Abstract

Point-of-care ultrasounds (US) are used during cardiopulmonary resuscitation (CPR) and after return of spontaneous circulation (ROSC). Carotid ultrasounds are a potential non-invasive monitoring tool for chest compressions, but their general value and feasibility during CPR are not fully determined. In this prospective observational study, we performed carotid US during conventional- and extracorporeal CPR and after ROSC with at least one transverse and coronal image, corresponding loops with and without color doppler, and pulsed-wave doppler loops. The feasibility of carotid US during (peri-)arrest and type and frequency of diagnostic findings were examined. We recruited 16 patients and recorded utilizable US images in 14 cases (88%; complete imaging protocols in 11 patients [69%]). In three of all patients (19%) and in 60% (3/5) of cases during CPR plus a full imaging protocol, we observed: (i) in one patient a collapse of the common carotid artery linked to hypovolemia, and (ii) in two patients a biphasic flow during CPR linked to prolonged low-flow time prior to admission and adverse outcome. Carotid artery morphology and carotid blood flow characteristics may serve as therapeutic target and prognostic parameters. However, future studies with larger sample sizes are needed.

## 1. Introduction

In Vienna, 42.6/100,000 inhabitants per year are treated after cardiac arrest (CA) and 11.3% of those survive to hospital discharge [1]. Rapid start of basic life support (BLS) and high quality of chest compressions are of utmost importance for favorable outcomes after CA [2,3]. However, chest compressions (CC) are highly susceptible to confounders such as tiring of providers; therefore, continuously monitoring CC quality is vital [4]. Carotid ultrasound (US) is currently seen as a potential non-invasive monitoring tool for CC efficacy [5,6]. Furthermore, it facilitates the detection of a pulse during rhythm checks [7,8]. The main objective of this study was to investigate the general feasibility of carotid US during CPR measures, as well as its potential as a novel therapeutic target.

## 2. Materials and Methods

Our main objective was to evaluate the general feasibility of carotid US during CPR, as well as the subsequent findings in 2D, color doppler, and pulsed-wave (PW) doppler imaging. We conducted a prospective observational trial in a convenience sample of patients admitted to our emergency department with an adherent high-volume certified Cardiac Arrest Centre in a tertiary care academic hospital. We included patients during conventional, LUCAS (Lund University Cardiopulmonary Assist System)-assisted CPR, extracorporeal CPR (ECPR), as well as patients immediately (<1 h) after return of spontaneous circulation (ROSC). Exclusion criteria consisted of: age <18 years, suspected pregnancy, traumatic CA, and potential neck or carotid injury.

As surrogate parameter for feasibility, we used the percentage of total patients in which we were able to acquire a complete imaging protocol.

Findings of interest were defined as unexpected observations with diagnostic or prognostic potential. The percentage of total cases in which image acquisition was feasible was recorded. As the common carotid artery (CCA) is an exposed and easily-accessible vessel, we chose it as the investigation site. All ultrasound examinations were conducted by an experienced team member of the cardiac arrest center using equipment by Phillips Healthcare (Epiq^®^ and Affiniti^®^). The investigations were conducted as follows: the probe (L12-3, linear, 12-3 MHz, Phillips Healthcare, Hamburg, Germany) was placed in the transverse plane over the sternocleidomastoid muscle (SCM) as far proximal as possible. The CCA was then identified in this image. Next, loops with and without color doppler were recorded. The probe was then rotated into the coronal plane. Another color doppler loop was recorded. For the pulsed-wave (PW)-doppler images, the probe was then tilted to achieve a measuring angle of 50–60°. PSV was determined as the point of the curve with the highest velocity, and EDV was measured right before the following systolic peak. If possible, we acquired bilateral images. Reasons for incomplete imaging protocols were termination of resuscitation before sufficient imaging material could be obtained, suboptimal measuring angle, and difficulties to obtain a valid PW doppler loop during ongoing LUCAS-assisted CPR. Furthermore, we acquired demographic parameters including age and sex, the mean arterial pressure (MAP), end-tidal carbon dioxide (etCO_2_), CA circumstances including place of CA, bystander CPR, duration of no-flow/low-flow, initial rhythm, blood gas analyses, and outcome (ROSC, survival to discharge). This study was approved by the Ethics Committee of the Medical University of Vienna (No. 1638/2018). Informed consent was acquired from all survivors before discharge and waived for non-survivors.

Continuous data were indicated as medians and interquartile ranges or means and standard deviations (SD), discrete data as counts and percentages. For comparisons between groups, we used the Kruskal-Wallis-Test. A *p*-value of <0.05 (2-tailed) was considered statistically significant. Microsoft Excel (Version 2013, Microsoft Corp., Redmond, WA, USA) and R-Studio (Version 1.4.1106, ©2009–2021 RStudio, PBC) were applied to conduct the analyses.

## 3. Results

From October 2018 to November 2019, 16 patients (61 (49–70) years; 69% male) were recruited; all but one suffered from out-of-hospital CA (OHCA). Half of the patients had an initially shockable rhythm. ROSC was achieved in 44%, and survival to hospital discharge could be achieved in 19% (Table 1).

**Figure 1:**
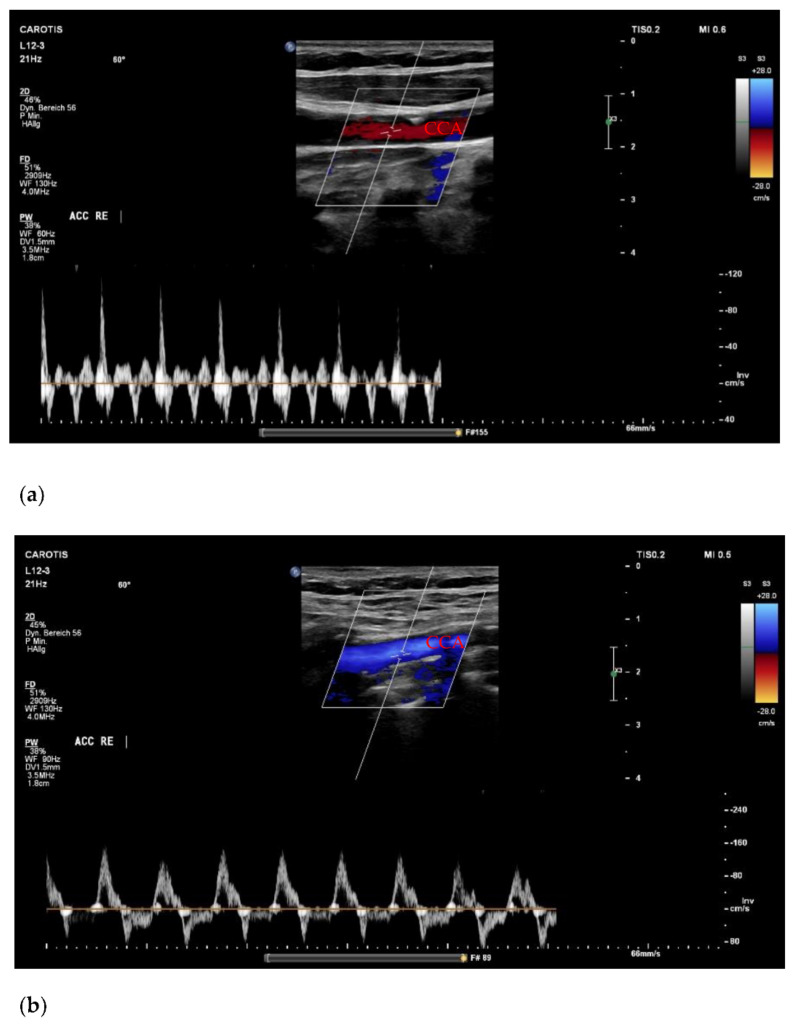
(**a**) Forward flow during compression/reverse flow during decompression in the CCA paired with small systolic peaks during conventional CPR; (**b**) Forward flow during compression/reverse flow during decompression with regular systolic peaks in a patient with transcatheter valve implantation. CCA = common carotid artery; CPR = cardiopulmonary resuscitation.

**Figure 2:**
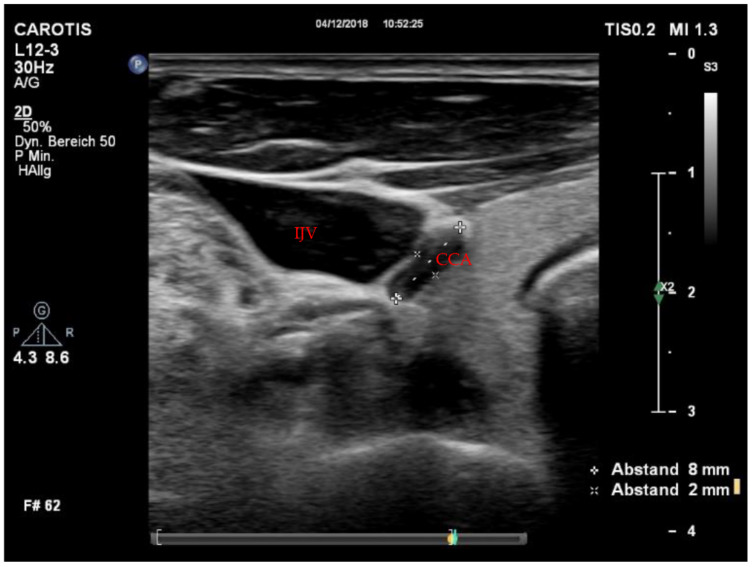
Collapse of the CCA during hypovolemic CA. CCA = common carotid artery; CPR = cardiopulmonary resuscitation; IJV = internal jugular vein.

Utilizable US images were acquired in 14 cases (88%), and we completed the full imaging protocol in 11 patients (69%). Of those, five (63%) were measured during conventional CPR, four (25%) immediately after ROSC, and two (12%) during ECPR. All patients with incomplete imaging protocols were assessed during conventional CPR. There were no significant differences in peak systolic velocity (PSV) or enddiastolic velocity (EDV) between CPR, ECPR and ROSC cases. However, a trend towards higher values of EDV during ECPR could be shown (Table 1). In the comparison of flow velocities, only participants with completed imaging protocols were included. Concerning potential findings of clinical value, we detected a forward-flow during compression with a reverse flow during decompression in two cases (Figure 1) and a collapse of the CCA in one case (Figure 2). Videos of images of interest can be found in the Supplemental Material.

## 4. Discussion

Point-of-care US (POCUS) is commonly used during CPR [9,10]. When practiced by a skilled provider, its use is positively associated with ROSC [11]. However, carotid US during CA is still a debated topic. In our investigation, we feasibly conducted carotid US while full advanced life support (ALS) was ongoing in most patients, and we observed pathologies of potential clinical interest. The latter are discussed in greater detail below.

### 4.1. Dynamics in Carotid Artery Flow

The noted forward flow during compression with reverse flow during decompression (Figure 1) has been previously observed using a non-imaging probe in a porcine CA model [12]. A similar pattern is associated with the diagnosis of cerebral circulatory arrest, for which an internal carotid ultrasound is commonly used and a highly reliable procedure [13]. However, this biphasic flow can also be present in the CCA, which was chosen as the imaging site in our study [14]. The therefore hypothesized cerebral circulatory arrest could be caused by early cerebral edema, further decreasing the cerebral perfusion pressure during CPR [15] and could be seen as a general sign of cerebral injury. In line with this, both images in Figure 1 were acquired from patients with prolonged low flow times prior to admission, in one case preceded by a long no-flow time. However, since the images were acquired from the CCA, it is possible that the PW doppler flow signal was altered by the vessels connected to the external carotid artery that are—as peripheral vessels—responsive to vasopressors and therefore “biased” in terms of wall tension and blood flow in a hyperadrenergic state [16] for instance occurring after the administration of high-dose adrenaline. In addition, one of the two patients had previously undergone a transcatheter aortic valve implantation (TAVI). Aortic valve insufficiency is a common occurrence after TAVI, and CPR is a known factor potentially leading to an implant failure [17,18]. This is of relevance since aortic valve insufficiency can produce a PW doppler signal similar to our findings [14]. If our results, however, reflect a certain degree or state of cerebral perfusion during CPR, the PW doppler signal and especially the EDV—preferably measured in the internal carotid artery—might be of future diagnostic and prognostic value. Naturally, further research is required to draw adequate conclusions.

### 4.2. Carotid Artery Collapse

A collapse of the CCA in a patient with hypovolemic CA was observed (Figure 2). Due to the very small diameter of the collapsed vessel, we were unable to acquire valid velocity measures—however, in past animal studies, a CCA collapse was detrimental to carotid blood flow [19]. Furthermore, it has been shown that higher blood flow velocities are associated with a higher invasive blood pressure [20]. In line with this, dynamics of the PSV in the CCA have been shown to be sensitive and specific to determine volume responsiveness in septic shock [21]. Similarly, we recorded a rise in PSV during ECPR after the additional initiation of continuous vasopressor support. These findings suggest that carotid US could be useful to determine hypovolemic origin of cardiac arrest and may guide volume resuscitation during CPR.

### 4.3. Comparison of Flow Velocities between CPR Modalities

To the best of our knowledge, this is the first human study that recorded carotid flow data from patients receiving various modes of CPR. During CPR, ECPR and after ROSC, PSV approached physiological values, comparable to previous studies investigating carotid US during conventional CPR [5,6]. The physiological values of EDV in the CCA amount to 20–30 cm/s [22], meaning that the median EDV during conventional, LUCAS-assisted CPR in our study was obviously lower and on average (mean EDV: 0 cm/second) not even present. This is mainly explained by the two patients with negative EDV and our small sample size. As expected, there was a trend towards higher EDV values during ECPR, which is of importance since diastolic flow is important to ensure a steady supply of blood throughout the cardiac cycle [23,24]. Of relevance, the mean EDV was very discriminative between groups, which, however, did not translate into statistically significant differences due to the very small group sizes. Further studies on flow velocities within and between CPR modalities with larger sample sizes are necessary, and should provide a more detailed insight on differences in cerebral perfusion. 

### 4.4. Limitations

Due to organizational reasons and the pilot study character of our investigation, only a very small sample size could be obtained, potentially influencing results. The generalizability of our findings is therefore not given. Findings might also be associated with the exact timepoint of assessment during or after CPR—certain pathologies may only occur after a certain period of time of low- or no-flow states. A potential further limitation is the measuring site, with the internal carotid artery potentially performing differently as the CCA. However, data on this problem are scarce and should be addressed in future research endeavors. Furthermore, as discussed in a recent study, Colour Doppler might be influenced by movement artefacts [25]. In our setting, this may be a substantial factor due to cardiac compressions. Another limitation relates to the unclear relationship between flow velocities measured in our study and the carotid artery blood flow (CABF). Of importance, an additional limitation in presenting ultrasound data is inter-observer differences, potentially also affecting carotid ultrasound during CPR. [26]

## 5. Conclusions

Changes in carotid artery morphology and carotid blood velocities during CPR are frequent and may serve as additional diagnostic and potential prognostic parameters.

## Figures and Tables

**Table 1:** Characteristics of study subjects.

**Baseline**	
Age, years (IQR)	65.0 (49.3–69.8)
Male sex, *n* (%)	11 (69%)
**CA details**	
OHCA, *n* (%)	15 (94%)
IHCA, *n* (%)	1 (6%)
Bystander CPR, *n* (%)	12 (75%)
EtCO2 (admission), mmHg (±SD)	31.4 (±26.5)
**Outcome**	
ROSC, *n* (%)	7 (43.8%)
Survival to hospital discharge, *n* (%)	3 (18.8%)
**Setting during imaging**	
Conventional CPR, *n* (%)	10 (62.5%)
ECPR, *n* (%)	2 (12.5%)
Post ROSC, *n* (%)	4 (25%)
**Low-flow time**	
Overall, minutes (IQR)	42.0 (23.0–62.0)
Deceased, minutes (IQR)	61.0 (38.3–63.5)
Sustained ROSC, minutes (IQR)	32.5 (19.35–48.5)
Survival to hospital discharge, minutes (IQR)	23.0 (13.5–36.0)
**Initial rhythm**	
VF, *n* (%)	7 (44%)
PVT, *n* (%)	1 (6%)
PEA, *n* (%)	7 (44%)
Asystole, *n* (%)	1 (6%)
**Blood gas analysis**	
PH (±SD)	6.84 (±0.24)
Lactate, mmol/L (IQR)	18.0 (10.0–19.0)
Base excess, mmol/L (IQR)	−19.8 (−23.7–−17.0)
**Carotid flow values**	
**PSV**	*p* = 0.880
CPR, cm/second (IQR)	84.4 (73.1–120.0), *n* = 5
ECPR, cm/second (IQR)	70.7 (62.0–79.3), *n* = 2
ROSC, cm/second (IQR)	85.0 (68.4–96.7), *n* = 4
**EDV**	*p* = 0.060
CPR, cm/second (IQR)	0.0 (−40.0–0.0), *n* = 5
ECPR, cm/second (IQR)	49.55 (47.63–51.47), *n* = 2
ROSC, cm/second (IQR)	28.6 (20.3–37.5), *n* = 4

OHCA = out-of-hospital cardiac arrest; IHCA = in-hospital CA; CPR = cardiopulmonary resuscitation; ECPR = extracorporeal CPR; (s)ROSC = (sustained) return of spontaneous circulation; VF = ventricular fibrillation; pVT = pulseless ventricular tachycardia; PEA = pulseless electrical activity; BE = base excess; PSV = peak systolic velocity; EDV = end diastolic velocity.

## Data Availability

Data are available from the corresponding author upon reasonable request.

## Supplementary Materials

The following are available online at https://www.mdpi.com/article/10.3390/jcm11020469/s1. Video S1. Transverse view of the CCA (image right) and IJV (image left). The CCA is collapsed independent of compression while the vein remains compressible. The cause of the patients CA was hypovolemia due to pulmonary bleeding. CCA = common carotid artery; IVJ = internal jugular vein; CA = cardiac arrest. Video S2. Coronal view of the CCA with colour doppler. The vessel shows forward flow during compression and reverse flow during decompression. Note that there are no signs of aliasing. CCA = common carotid artery Video S3. Coronal view of the CCA with colour doppler. The vessel shows continuous forward flow with minimal variation. The loop was recorded shortly after the patient regained circulation during eCPR. CCA = common carotid artery.
